# Social Isolation and Healthcare Utilization Among Older Adults Living in Subsidized Housing

**DOI:** 10.1093/geroni/igab046.523

**Published:** 2021-12-17

**Authors:** Thomas Cudjoe, Laura Prichett, Katherine Runge, Laura Andes, Carl Latkin, Cynthia Boyd

**Affiliations:** 1 Johns Hopkins University School of Medicine, Baltimore, Maryland, United States; 2 Johns Hopkins BEAD, Baltimore, Maryland, United States; 3 Mercy Housing, Denver, Colorado, United States; 4 Johns Hopkins Bloomberg School of Public Health, Baltimore, Maryland, United States; 5 Johns Hopkins University, Baltimore, Maryland, United States

## Abstract

Older adults living in subsidized housing are often at high risk for having multiple chronic conditions and nursing home placement. Previous studies in this population have not examined the relationship between social isolation and healthcare utilization. We examine this using Lubben Social Network Scale-6 and self-reported healthcare utilization. Utilizing data from a multi-state non-profit subsidized housing provider, we performed descriptive and multivariate analyses on a sample of older adults (N=3,822). Overall, 95 % reported having a checkup within the last 12 months and an average of less than one emergency room visits (mean= 0.58) or hospitalizations (mean= 0.34). In adjusted models, Socially isolated older adults had lower levels of routine checkup (OR=0.50, CI 0.36,0.70) and higher levels of hospitalizations (IRR=1.30, CI 1.10,1.54) compared to older adults who were not socially isolated. Efforts to address healthcare utilization should identify social isolation and explore strategies to promote social connectedness to improve health.

